# Beat-to-Beat Cycle Length Variability of Spontaneously Beating Guinea Pig Sinoatrial Cells: Relative Contributions of the Membrane and Calcium Clocks

**DOI:** 10.1371/journal.pone.0100242

**Published:** 2014-06-18

**Authors:** Massimiliano Zaniboni, Francesca Cacciani, Robert L. Lux

**Affiliations:** 1 Department of Life Sciences, University of Parma, Parma, Italy; 2 Center of Excellence for Toxicological Research, University of Parma, Parma, Italy; 3 Cardiovascular Research and Training Institute, University of Utah, Salt Lake City, Utah, United States of America; Brigham & Women’s Hospital - Harvard Medical School, United States of America

## Abstract

The heartbeat arises rhythmically in the sino-atrial node (SAN) and then spreads regularly throughout the heart. The molecular mechanism underlying SAN rhythm has been attributed by recent studies to the interplay between two clocks, one involving the hyperpolarization activated cation current I_f_ (the membrane clock), and the second attributable to activation of the electrogenic NaCa exchanger by spontaneous sarcoplasmic releases of calcium (the calcium clock). Both mechanisms contain, in principle, sources of beat-to-beat cycle length variability, which can determine the intrinsic variability of SAN firing and, in turn, contribute to the heart rate variability. In this work we have recorded long sequences of action potentials from patch clamped guinea pig SAN cells (SANCs) perfused, in turn, with normal Tyrode solution, with the I_f_ inhibitor ivabradine (3 µM), then back to normal Tyrode, and again with the ryanodine channels inhibitor ryanodine (3 µM). We have found that, together with the expected increase in beating cycle length (+25%), the application of ivabradine brought about a significant and dramatic increase in beat-to-beat cycle length variability (+50%). Despite the similar effect on firing rate, ryanodine did not modify significantly beat-to-beat cycle length variability. Acetylcholine was also applied and led to a 131% increase of beating cycle length, with only a 70% increase in beat-to-beat cycle length variability. We conclude that the main source of inter-beat variability of SANCs firing rate is related to the mechanism of the calcium clock, whereas the membrane clock seems to act in stabilizing rate. Accordingly, when the membrane clock is silenced by application of ivabradine, stochastic variations of the calcium clock are free to make SANCs beating rhythm more variable.

## Introduction

The spontaneous beating activity of the heart is characterized by cycle length variability between consecutive beats, the so called heart rate variability (HRV), which is under primary control of the autonomic nervous system [1]. The complexity related to HRV is required for proper functioning of the cardiac pump, and a wide spectrum of pathological conditions arises when such complexity is lost [2]–[6]. A certain degree of beat-to-beat variability in the rate of firing is intrinsically present not only at the level of isolated (Langendorff perfused) heart [7], but also within the isolated sino-atrial node (SAN), the natural cardiac pacemaker [8], and even at the level of single cells enzymatically isolated from SAN [6], [9], [10]. Although it is known that this latter form of variability is modulated by electrotonic interaction within the surrounding tissue [11]–[13], nonetheless it is relevant to measure it in the various species, since it has been suggested that, in the setting of cardiac disease, a partial breakdown of autonomic control may unmask such intrinsic cellular component of HRV [14].

A first account of the extent of the intrinsic irregularity in the beating rate of single pacemaker heart cells was provided by Wilders and Jongsma [9], who measured a 2.0% coefficient of variability for their inter-beat interval (i.e. cycle length CL). HRV in adult unrestrained rabbits is about 10% [15]. Similar findings are also available for different preparations, including those in spontaneously beating clusters of embryonic chick ventricular cells, single neonatal atrial and ventricular cells, and small groups of neonatal rat heart cells [11], [12]. Using computer simulations, Wilders and Jongsma [9] demonstrated that beat-to-beat variability of CL in rabbit SANCs can be well described in terms of stochastic open-close kinetics of membrane ion channels. They also showed that inter-beat variability of CL in isolated SANCs tends to be normally distributed, and that consecutive CLs do not correlate over a lag of 1–20 beats. Furthermore, as pointed out by Rocchetti et al. in isolated rabbit SANCs exposed to ACh or isoproterenol, CL variability is expected to increase with mean CL, simply due to the hyperbolic-like relationship between CL and diastolic depolarization rate [16], [17]. Since the main mechanism underlying SAN automaticity has been historically recognized to be the hyperpolarization activated I_f_ current [18]–[22], it is straightforward to think this current to be involved in the mechanism underlying inter-beat CL variability, and to view it as a possible target for modulating such variability.

Recently, it has been proposed that, in addition to I_f_ serving as a so-called “membrane clock”, rhythmic release of SR calcium also contributes, as a “calcium clock”, to SA nodal diastolic depolarization (DD) [23]. The Ca^2+^ clock mechanism operates by generating spontaneous local subsarcolemmal Ca^2+^ releases (LCRs) during late DD, which activate forward Na^+^-Ca^2+^ exchange, providing a cyclic source of depolarizing current [24]–[26]. The stability and flexibility of pacemaker function likely depends on the synergistic interplay between the two clocks [23]. Since the very nature of LCRs that sustain the calcium clock is stochastic, it has been shown recently that their beat-to-beat variations sustain spontaneous beat-to-beat variability of CL in single SANCs [10].

Despite their relevance, the relative roles of both clocks in determining intrinsic beat-to-beat variability of SANCs firing has never been dissected in detail. In the present study we measured beat-to-beat CL variability in sequences of spontaneous action potentials (APs) recorded from enzymatically isolated guinea pig SANCs. We measured the same parameter during pharmacological interventions which were expected to inhibit, in turn, the two clocks, i.e. by exposing the cells to ivabradine, a specific I_f_ blocker, and to ryanodine, a specific SR release blocker. Our results suggest for the first time that the calcium clock provides a major contribution to beat-to-beat CL variability, as compared to the membrane clock, which is extremely relevant for understanding both the physiology and pharmacology of sinus rhythm.

## Materials and Methods

### Cell Isolation

#### Ethics Statement

The experimental procedure was approved by the Veterinary Animal Care and Use Committee of the University of Parma (Prot. N° 41/11) and carried out in strict accordance with the National Ethical Guidelines (Italian Ministry of Health; D.L. vol. 116, January 27, 1992).

The isolation protocol was adapted from [20]. Male Dunkin-Hartley guinea pigs, weighing 300–350 g, were anesthetized by ether inhalation and killed by decapitation. The heart was quickly removed and placed in a beaker containing 60 mL of solution 1 containing 500U heparin, rinsed in the same solution in order to remove blood, and pinned, exposing the anterior ventral face, to the silicon bottom of a plexiglass chamber filled with solution 1 at 35°C (for solutions composition, see below). After removing the ventricles, the anterior wall of the right atrium was split and the posterior wall exposed in order to identify the SAN region, which was then dissected and minced in tiny fragments. These were then transferred into a beaker with solution 1 at 35°C, and gassed with oxygen 100% for 50 minutes. They were then washed in solution 2 and underwent enzymatic digestion in solution 2 containing 2 mg/ml Type I Collagenase (Worthington, Lakewood, NJ, USA), 0.1 mg/ml Elastase (Sigma-Aldrich, Milan, Italy) and 1 mg/ml Albumin for 25 minutes. After a second passage in solution 2 without enzymes, tissue underwent a further phase (15 min) of mechanical dispersion in solution 3.

Calcium concentration was then increased from 0 to 0.8 mM in a 4-step (9 min) process by addition of increasing volumes of solution 1. Cells were then allowed to settle for about 30 minutes, after which supernatant was removed and replaced with solution 1. Cells where used for electrophysiological measurements within the next 6–8 hours.

### Solutions

Solution 1: (in mM): NaCl 140; KCl 5.4; MgCl_2_ 1; CaCl_2_ 1.8; Glucose 10; HEPES 5; pH 7.4 with NaOH. Solution 2: NaCl 140; KCl 5.4; MgCl_2_ 0.5; KH_2_PO_4_ 1.2; Taurine 50; Glucose 10; pH 7 with NaOH. Solution 3: KCl 20; KH_2_PO_4_ 10; Glutamic acid 70; β-Hydroxybutyric acid 10; Taurine 10; Glucose 10; Albumin 1 mg/ml; HEPES 10; pH 7.4 with KOH. Solution 4 (also referred to as normal Tyrode, NT): NaCl 140; KCl 5.4; MgCl_2_ 1; CaCl_2_ 1.8; Glucose 10; NaH_2_PO4 1; HEPES 5; pH 7.4 with NaOH. Solution 5: NaCl 10; KCl 113; MgCl_2_ 0.5; Glucose 5.5; NaH_2_PO4 1; K_2_ATP 5; HEPES 10; pH 7.1 with KOH.

### Electrophysiological Measurements

Cells in solution 1 were placed in the perfusion chamber of an inverted microscope (Nikon Eclipse T300), allowed to settle for 10 minutes, and then perfused at 36°C with solution 4. Patch pipettes were made from borosilicate capillaries (Clark capillaries, Harvard Apparatus LTD, Edenbridge, UK) by means of a two-step vertical puller (Narishige PC-10, Narishige, Japan) and had a tip resistance, when filled with solution 5, of 2–5 MΩ. Transmembrane potential (V_m_) was recorded by means of an Axoclamp 2B amplifier (Molecular Devices, Sunnyvale, CA) adopting the whole-cell configuration of the patch clamp technique, and digitized at a sampling rate of 10 kHz with a 12-bit A/D converter (Digidata 1200, Molecular Devices, Sunnyvale, CA). Pipette potential was set to zero and tip resistance was compensated by bridge-balance before contacting cell membrane. Spontaneously beating cells were brought in patch-clamp whole-cell configuration, and series of action potentials (AP) recorded via the pCLAMP6 software (Molecular Devices, Sunnyvale, CA), under control conditions (solution 4), until the AP configuration reached a steady-state. Perfusion solution was then rapidly switched, by means of a 6-way electro-valve (Cole-Parmer, General Control, Milan, Italy) to a number of different solutions, and the recording protocol repeated. Test solutions were obtained by adding solution 4, in turn, with: acetylcholine (ACh) 10 nM, isoproterenol (Iso) 100 nM, ivabradine (Iva, Sequoia Research Products Ltd, Pangbourne, UK) 3 µM, or ryanodine (Rya, Ascent Scientific Ltd, Bristol, UK) 3 µM. Perfusion of test solutions could last from 3 to 5 minutes, depending on the time needed to reach a new steady state AP configuration. All solutions were kept at 36°C before and during perfusion.

### Data Analysis

**Figure 1:**
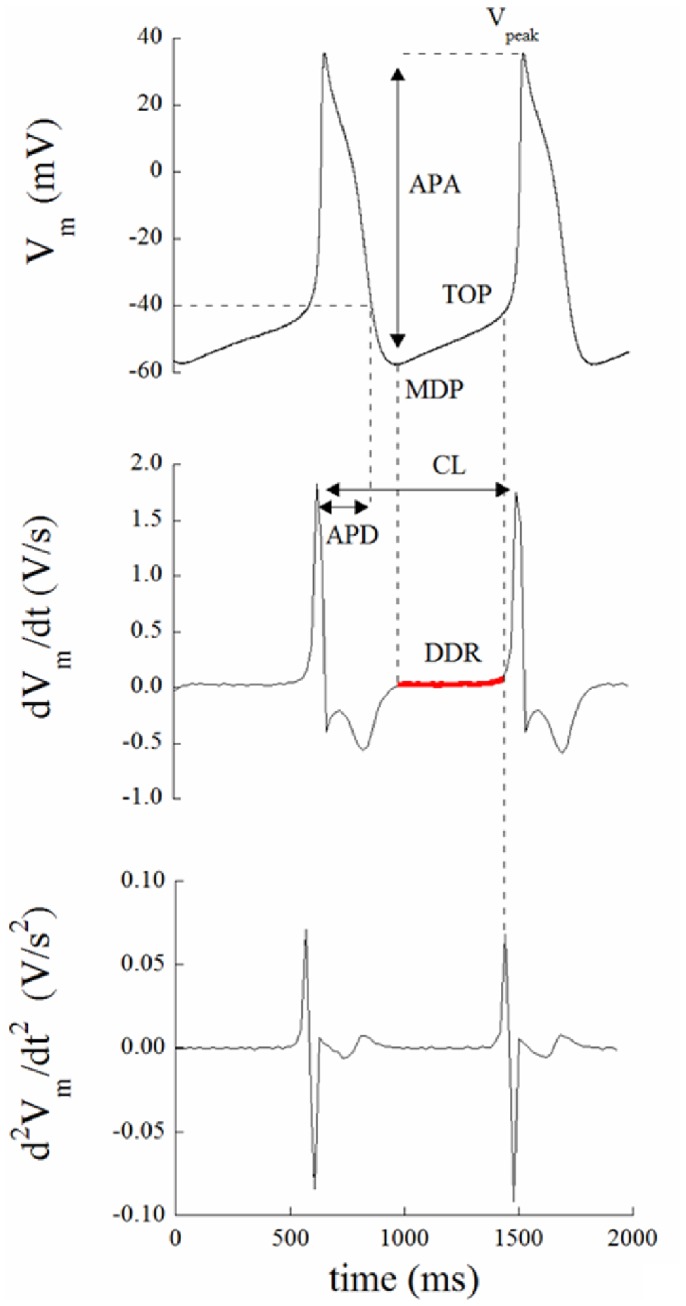
Action potential parameters measurement. Shown are APs (top), first and second time derivatives (middle and bottom, respectively). For each beat, the following parameters were automatically measured: MDP (maximum diastolic potential, mV), as the most negatively polarized value reached by membrane potential during each cycle; V_peak_ (mV), as the most positively polarized value reached by membrane potential during each cycle; APA (action potential amplitude, mV), as the difference between the two preceding parameters; TOP (take-off potential, mV), as the V_m_ taken at the time of the peak of the second derivative of V_m_ with respect to time; DD (diastolic depolarization phase, ms), as the time interval between MDP and TOP; DDR (diastolic depolarization rate, V/s), as the average value of the first derivative of V_m_ over DD (marked in red in the middle panel); APD (action potential duration, ms), as the time between the peak of the first derivative of V_m_ and the time when V_m_ reaches the value of −40 mV.

Data were analyzed by means of a custom software written by co-author R.L. Lux (ScalDyn) running on an Apple MacBook Pro 2.5 GHz, and of a dedicated software written in Matlab (The MathWors, Inc., USA). Long sequences of spontaneous APs were then analyzed to measure the parameters described in Figure 1. Scaldyn algorithms used first and second least mean squared parabolic estimates of first and second derivatives to detect beats, identify AP fiducials (AP onset, peak and MDP times) from which AP amplitudes, intervals and rates were determined. The program detected beats and allowed stacking and scanning of consecutive beats to visualize intervention-induced changes to AP waveform and CL variation. Mean values of parameters and their coefficient of variability (CV = SD/mean*100) were calculated over 30 s sequences of consecutive beats. Beat-to-beat CL variability was also quantified from Poincarè plots representations of CL sequences as:
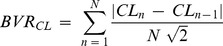
where N is the total number of beats of the sequence, and CL_n_ the n^th^ element of it [27].

### Statistical Analysis

Data are presented as mean ± SE. Paired data Student’s t tests were performed in order to assess the effect of blockers. Frequency distributions and autocorrelation analysis were performed in Matlab.

## Results

### Modulation of Membrane and Calcium Clock

**Figure 2:**
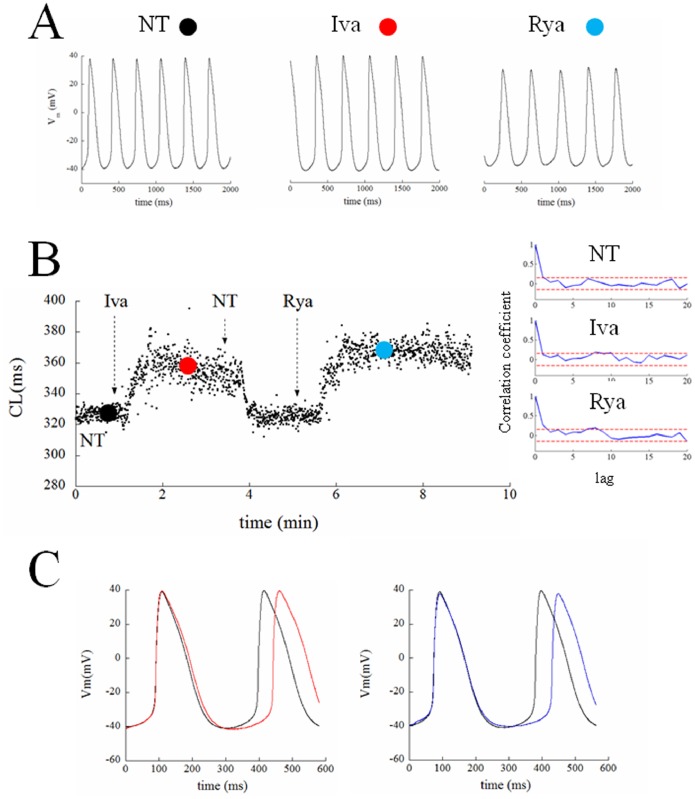
Chronotropic effect of ivabradine and ryanodine. Panel A: Series of APs were recorded from a patch clamped SANC, first perfused in NT, then in 3 µM ivabradine, then back in NT (not shown), and finally in 3 µM ryanodine. Switching times are reported as vertical arrows. Time was allowed for AP parameters to reach the steady state in each perfusion condition, and 2 s samples of each sequence were taken and reported here. Panel B: The time course of CL of the entire experiment is reported on the left. Colored dots represent the times when the 2 s sequences of Panel A were taken. Three sequences of 158 beats were taken from the three regions ending with the colored dots, and underwent autocorrelation analysis of lags of 1–20 beats; results are shown in right Panel. C: Representative examples of the observed bradycardic effect of ivabradine (left) and ryanodine (right) on APs (black: controls; red: ivabradine; blue: ryanodine).

**Figure 3:**
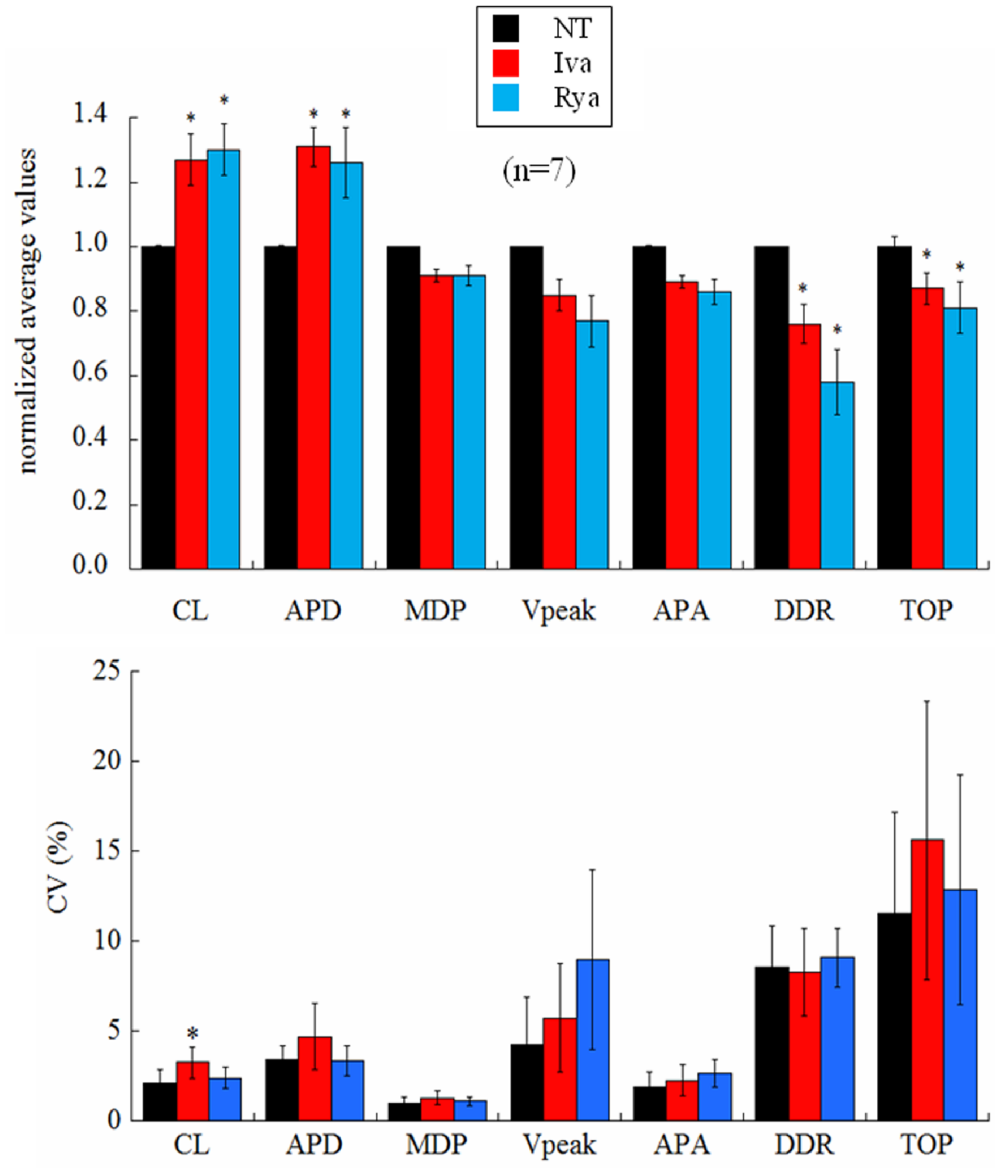
Effects of ivabradine and ryanodine on AP parameters. (Top) The histogram summarizes changes induced on AP parameters by ivabradine (red) and ryanodine (blue) over 30 s sequences after beating reached steady state conditions. All values are reported as normalized with the corresponding mean value measured in NT (black). (Bottom) Corresponding percent changes in beat-to-beat variability of AP parameters, measured as CV. Statistics: paired t-test p<0.05 (*).

**Figure 4:**
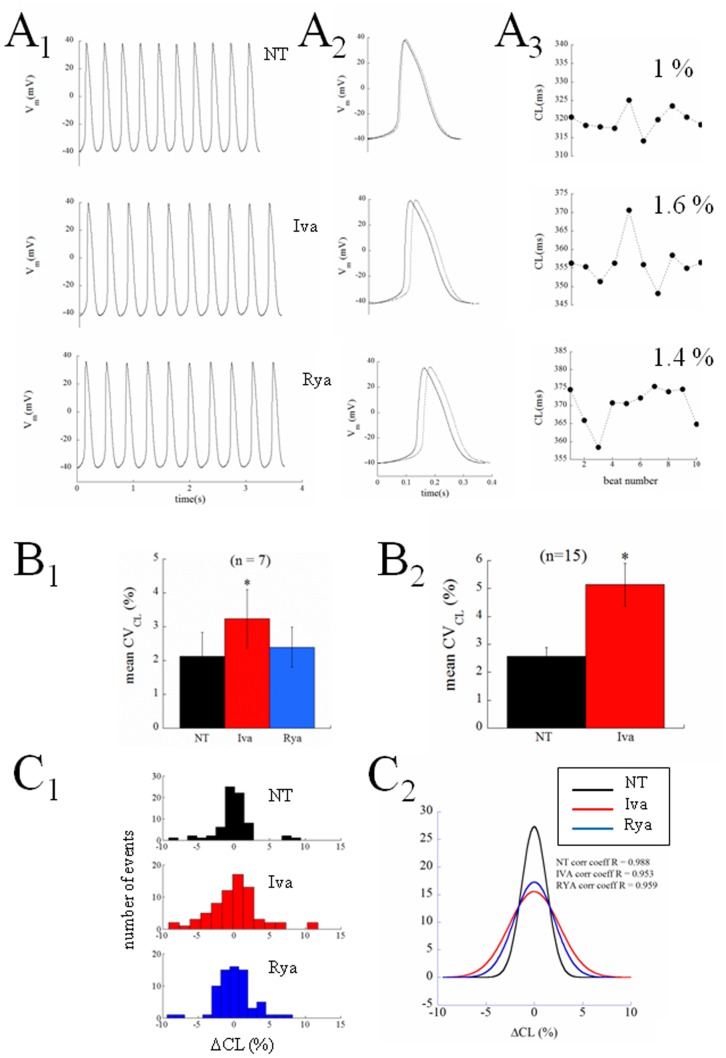
Beat-to-beat variability of CL: percent changes. The same protocol described in Figure 2 was performed on another cell. A_1_: 10-beats sequences recorded in the three perfusion conditions. A_2_: superimposed shortest (NT) and longest (treated) CLs, referenced to the preceding beat, taken from each sequence. A_3_: time course of CL over the corresponding 10 beats. Percent values are CV_CL_ of each 10 beats sequence. B_1_: Seven cells underwent the entire protocol; the average CV_CL_ measured on this sample is reported in the histogram. B_2_: On a larger number of cells we only succeeded in applying NT and ivabradine; mean CV_CL_ is reported in the histogram for this larger sample. C_1_: all seven 10-beats CL sequences for the 3 perfusion conditions, each one normalized for the corresponding average value of CL, are pooled together in the 15-bins frequency histograms. All frequency distributions were well fitted by Gaussian curves, which are reported in Panel C_2_ with the corresponding correlation coefficients. Statistics: paired t-test p<0.05 (*).

**Figure 5:**
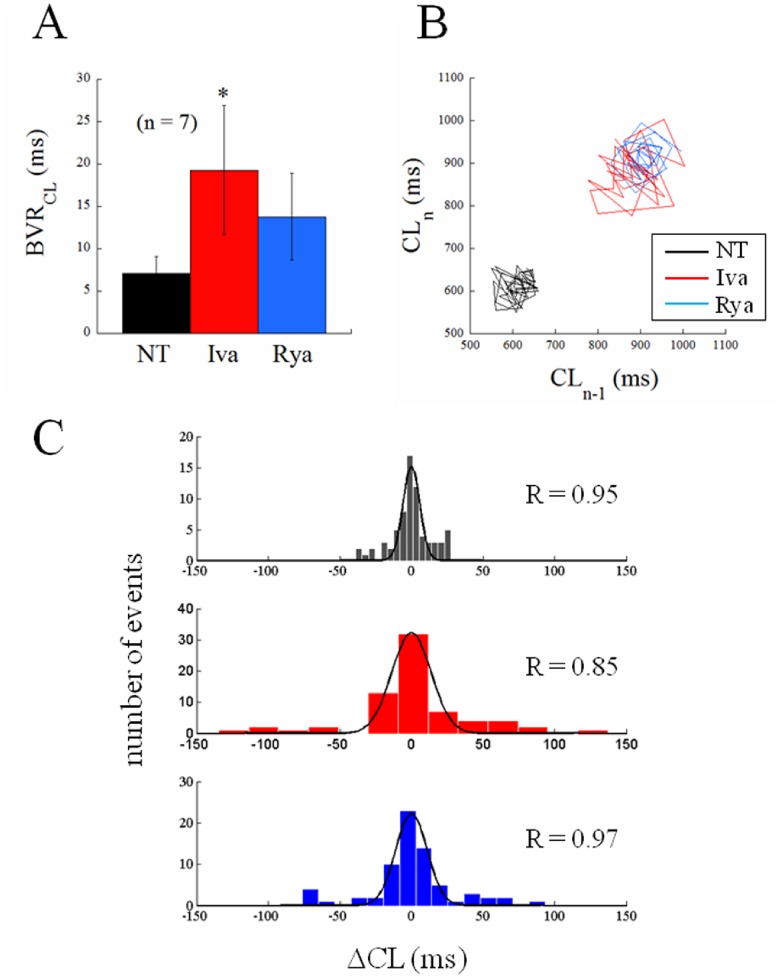
Beat-to-beat variability of CL: absolute changes. The inter-beat variability of the same CL sequences analyzed in Figure 4 in terms of CV (%) were also analyzed in terms of BVR (ms, see Methods). A: average BVR values are reported for the AP sequences recorded in steady state conditions for the 3 different treatments. B: representative example of Poincarè plot for 30 s sequences recorded from the same cell in the 3 experimental conditions after beating rate reached its steady state. C: all seven 10-beats CL sequences for the 3 perfusion conditions reported in panel A were pooled together in the 15-bins frequency histograms. The frequency distributions were well fitted by the superimposed Gaussian curves; fitting correlation coefficients are reported in each panel. Statistics: paired t-test p<0.05 (*).

**Figure 6:**
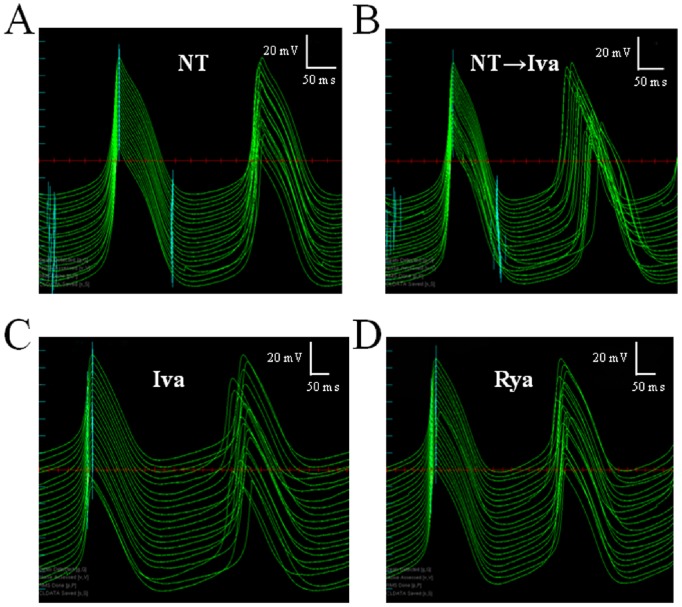
Scrolling through AP sequences with ScalDyn. One of the features of the software ScalDyn is the ability to time-align and stack single, pairs or multiple consecutive APs (with respect to the time of their maximum value of dV_m_/dt) of a long sequence, and scroll through the entire recording back and forth. The panels in figure show 20 consecutive pairs of APs recorded from the same cell in different conditions; in each panel APs on the left are synchronized as explained above, whereas those on the right (the succeeding AP for each preceding AP) show average CL changes and beat-to-beat CL variability. Variability in NT is low (A) throughout the entire perfusion time. CL prolongation is evident in B, where APs recorded during the switch between NT and ivabradine are shown (top to bottom). Panel C shows steady state condition with ivabradine displaying the highest level of CL beat-to-beat variability. Steady state condition in ryanodine (D) shows some beat-to-beat variability, though visually much less than in ivabradine.

The protocol that we applied to each patch clamped guinea pig SANC is illustrated in Figure 2. All patched cells of this study showed spontaneous regular contractions. After AP waveform stability was reached, spontaneous electrical activity was recorded for about 2 min under perfusion of NT solution; perfusion medium was then rapidly switched to a solution containing the I_f_ inhibitor ivabradine (Iva, 3 µM) for approximately 3 min, then turned back to NT for 2 min, and then again to a NT combined with the SR release channel inhibitor ryanodine (Rya, 3 µM) for 3 min or more. Both inhibitors, at these concentrations, have been previously used to dissect the contribution of I_f_ and SR Ca^2+^ release to SANC firing rate [28]. In particular, micromolar concentrations (<10 µM) of ryanodine are known to force ryanodine channels into an open sub-conducting state, which leads to the abolishment of SR Ca^2+^ release and Ca^2+^ transient [29], [30]. In some cells the seal was lost before the entire sequence of solutions was applied, some did not recover their initial AP configuration after returning in NT from Iva perfusion, while others spontaneously depolarized throughout the experiment. This resulted in a successful application of the entire protocol to only 7 cells, whose data analysis is reported in Figures 2 to 6. Figure 2A shows three typical AP sequences obtained from the same cell at steady state in control condition (NT), during I_f_ inhibition (Iva), and during SR release inhibition (Rya). An example of the sequence of measured CL of the spontaneous APs during this experiment is illustrated in left panel B. Panel C shows representative examples on how Iva and Rya (red and blue traces respectively) modified the intrinsic AP shape (black line) of this cell. A number of 158 beats was taken from each treatment right before the switch to the next solution and underwent calculation of serial correlation coefficient of lag 1–20 beats (Panel B on the right). No autocorrelation was found (confidence level 0.05, red dotted horizontal lines in each Panel) in any of the three analyzed conditions. A similar result was found in the remaining 6 cells. The autocorrelation test shows that, at least within a 20 beats range, there are not periodic patterns hidden under the beat-to-beat CL variability.

Figure 3 (top) shows paired statistics for all cells. Iva application (red columns) led to a significant 27% prolongation of CL, 31% APD, a 24% decrease of DDR, and a 13% depolarization of TOP. Rya application (blue bars) led to a significant 30% prolongation of CL, 26% prolongation of APD, a 42% decrease of DDR, and a 19% depolarization of TOP. Actual mean values are reported in Table 1. Bottom panel of the same figure shows corresponding beat-to-beat variability, measured as coefficient of variability CV for each AP parameter.

**Table 1:** Summary of the effect of Iva and Rya on AP parameters.

	NT	Iva	Rya
CL (ms)	456.6±40.4	578.4±82.3	587.5±76.9
APD (ms)	164.5±7.2	219.6±24.5	211.5±20.0
MDP (mV)	−49.6±3.5	−45.9±4.3	−46.2±4.2
V_peak_ (mV)	29.6±2.7	26.1±4.2	23.3±4.4
APA (mV)	79.4±4.8	72.0±7.6	69.6±6.6
DDR (V/s)	0.09±0.02	0.07±0.02	0.06±0.02
TOP (mV)	−26.0±5.0	−23.2±5.6	−22.8±5.1

The actual mean values (n = 7) of AP parameters following application of Iva and Rya are reported, together with parameter values measured in NT. The same measurements, normalized to NT values, are shown in Figure 3.

### Beat-to-beat CL Variability

Figure 4 presents another example of the complete protocol described above on a different cell. Ten beats of the three sequences, NT, Iva, Rya, are shown in Panel A_1_. The longest and shortest AP cycles from each experimental condition are superimposed in Panel A_2_ and the entire corresponding time courses of CLs are given in Panel A_3_. Spontaneously beating SANCs show beat-to-beat CL variability that significantly increased after application of Iva and did not after application of Rya, even though both treatments achieved their expected and similar bradycardic effect. In Panel B_1_ we pool together and compare the coefficient of variability (CV_CL_) measured in 10 beats sequences of the n = 7 cells which underwent the entire protocol. Spontaneously beating SANCs displayed an intrinsic CV_CL_ = 2.12±0.71, this significantly increased up to a value of 3.24±0.86 during exposure to Iva (+52%), and did not undergo significant changes under exposure of Rya (2.39±0.59). As noted above, on several cells we only succeeded in partially applying the protocol; in many cases we could record sequences of APs in NT, in Iva, and, frequently, back in NT, without being able to apply Rya. We can therefore report statistics of the increase of CV_CL_ in Iva on a much larger sample (Panel B_2_), and see that, in this case, CV_CL_ changes from the intrinsic 2.58±0.31 up to the value of 5.15±0.76 in Iva (+100%).

Another way to look at these same differences is through the frequency distributions on the three histograms of Panel C_1_, where 7x10 beats sequences, each normalized to its average value, were pooled together and reported in 15 bins distributions for NT, Iva, and Rya. Such distributions were well fitted by Gaussian curves, which again show larger dispersion in the case of Iva treatment (Panel C_2_). A further proof that the AP sequences analyzed in Figure 4 in terms of CV were indeed at their pacing steady state is provided by the analysis of their Poincarè plots, which is summarized in Figure 5. Beat-to-beat CL variability, when analyzed as BVR_CL_ (see Methods), shows in fact the same qualitative behavior of CV_CL_ (compare Figure 5A and 5C with Figure 4B_1_ and 4C_1_). The absence of relevant asymmetries between the longitudinal and transverse components of Poincarè plots [27], [31], [16] is evident in the representative example of Figure 5B, which also clearly shows the same bradycardic effect of Iva and Rya, and their quite different effect on inter-beat CL variability. The different degree of beat-to-beat variability of CL is particularly evident in plots like those shown in Figure 6, where the analysis and measurement program, ScalDyn, allows stacking and scrolling through the entire sequence of APs recorded for one cell during the experiment. A common feature of the 7 cells analyzed is that, as already described above and made more clear here, beating rate is very stable under NT perfusion (Panel A), becomes more variable under Iva perfusion (Panels B and C), and doesn’t change significantly under Rya perfusion (Panel D). The NT, Iva, and Rya sequences reported in Figure 6 A, C, and D were all taken in steady state AP conditions.

The results presented so far suggest that a much higher degree of stochastic beat-to-beat CL variability is associated with the calcium clock, which is normally damped by the voltage-dependent activation of the membrane clock. Consequently, when the latter is inhibited, CL variability increases. Some smaller stochastic behavior is associated also with the membrane clock, as it is expected also from other ion currents [9], [32] underlying SANCs beating. This “control” variability is what is left after inhibiting the calcium clock.

### CL Dependency of CL Variability

**Figure 7:**
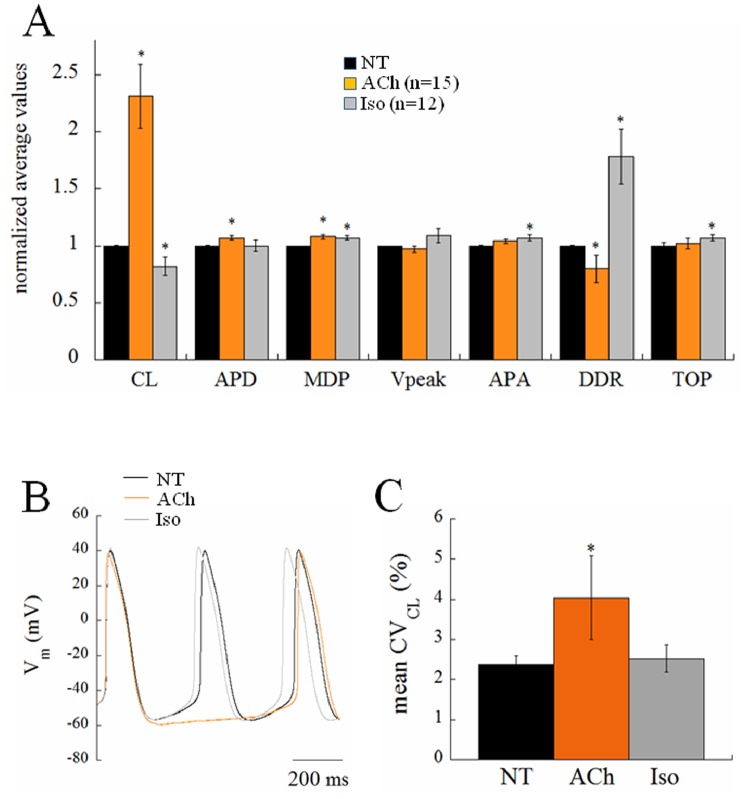
Chronotropic effect of acetylcholine and isoproterenol. Histogram in panel A summarizes the effects of acetylcholine (ACh) and isoproterenol (Iso) on AP parameters. Representative examples of the AP response to both agonists are shown in B. Panel C summarizes changes in beat-to-beat CL variability on a subgroup of n = 8 cells which underwent both treatments sequentially.

Previous literature [16] has shown that an increase in CL variability with CL is expected from a simple numerical relationship between AP parameters, which covers control, ACh, and Iso results. In order to verify this relationship within our experimental preparation, we exposed the cells to muscarinic agonist ACh (10 nM) and to the β-adrenergic agonist Iso (100 nM), respectively known to have negative and positive chronotropic effects on spontaneous beating rate of cardiac pacemaker cells (Figure 7). Indeed, Iso led to a significant decrease in average CL (−18%), essentially mediated, among other mechanisms, by a dramatic increase in DDR (+78%), whereas ACh led to a significant increase in average CL (+131%), likely mediated by increase in APD (+7%) and decrease in DDR (−20%). Autocorrelation analysis of the type shown in Figure 2B was also performed on long series of APs under ACh and Iso perfusion and no significant autocorrelation was found in either instance. The Iso-induced modest CL shortening did not lead to any significant change in CV_CL_, whereas the ACh-induced CL prolongation brought about a 70% increase of CV_CL_ (Figure 7C). Such an increase is slightly larger but within the range documented by Rocchetti et al [16].

**Figure 8:**
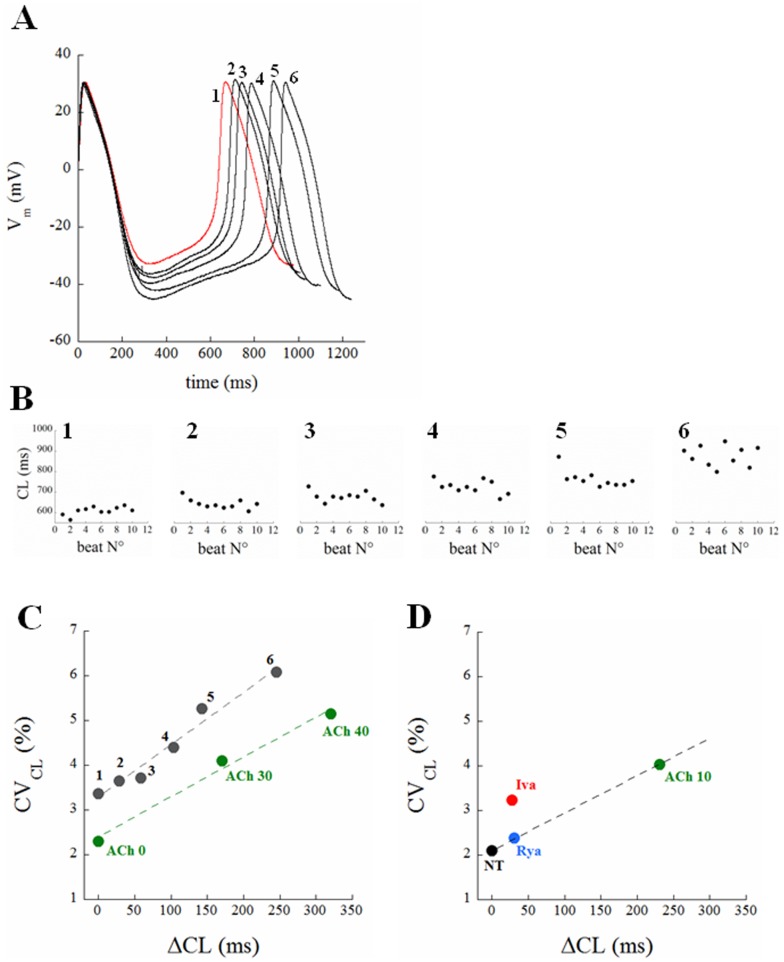
Correlation between CL and CV_CL_. Panel A: a steady AP waveform from a spontaneously beating single SANC is shown in red (trace 1). The cell was injected with constant hyperpolarizing currents of progressively increasing amplitude, i.e. 0.01, 0.02, 0.03, 0.04, 0.05 nA, each one lasting for 20 s in order to allow AP parameters to reach a new steady state. An AP cycle from the last 10 beats of each 20 s recording was taken and all reported superimposed in panel A (traces 2 to 6). Panel B: time course of CL for the last 10 beats corresponding to each current injection. ΔCL and CV_CL_ values of data in Panel B are summarized in the black dots of Panel C, which are reported together with the linear fit through them. ΔCL with respect to NT and corresponding CV_CL_ at 3 different acetylcholine concentrations (nM) were taken from [16] and also reported in Panel C (green dots). Panel D summarizes results from Figures 4 and 5. The beat-to-beat CL variability associated with each treatment is shown versus the corresponding average induced ΔCL.

In addition to modulating CL with chronotropic agents, we also used hyperpolarizing current injection to slow spontaneous firing. Figure 8A illustrates the results from a spontaneously beating cell subjected to 20 sec of hyperpolarizing current at five different amplitudes (from 10 to 50 pA, step 10). As the cell hyperpolarized, both CL and CV_CL_ increased (panel B). The results from this example are summarized in panel C (black dots), which shows a linear relationship between the average value of CL changes from the last 10 beats of each sequence (shown in detail in Panel B) and the corresponding CV_CL_. Also included in panel C are results from Rocchetti et al [16] (from figures 2 and 4 of their paper), who found a similar linear relationship between CV_CL_ and ΔCL induced by ACh (green dots for 0, 30, and 40 nM respectively).

Figure 8D summarizes the CV_CL_ versus ΔCL relationship for our results, including control conditions (NT), Rya (blue), ACh (green) and Iva (red). Rya induces a significant increase of CL with a slight (and not statistically significant) increase of CV_CL_. Similarly, ACh increases CL as well as CV_CL_ up to 4%. Interestingly these changes in CL and CV_CL_, when considered together with control values (NT, black dot), correlate linearly, as those shown in Panel C. Also, although Iva induces a ΔCL almost identical to that induced by Rya, the corresponding I_f_ blockade produces an increase in CV_CL_ which is much larger than the one expected from such correlation.

In other words, a certain extent of stochastic electrical noise is likely associated with most of ion channel kinetics throughout DD and is reflected into increase of CL dispersion as DDR decreases, and therefore CL prolongs. While most of whole-cell voltage-dependent ion currents, specifically I_f_, are intrinsically deterministic and occasionally stochastic, the calcium clock mechanism is intrinsically stochastic [10]. Accordingly, a fully expressed membrane clock offsets such variability (black dot in Panel D), whereas membrane clock inhibition increases it above the linearly correlating values expected from ion channel kinetics (red dot in the same Panel).

### CL vs DDR Relationship

**Figure 9:**
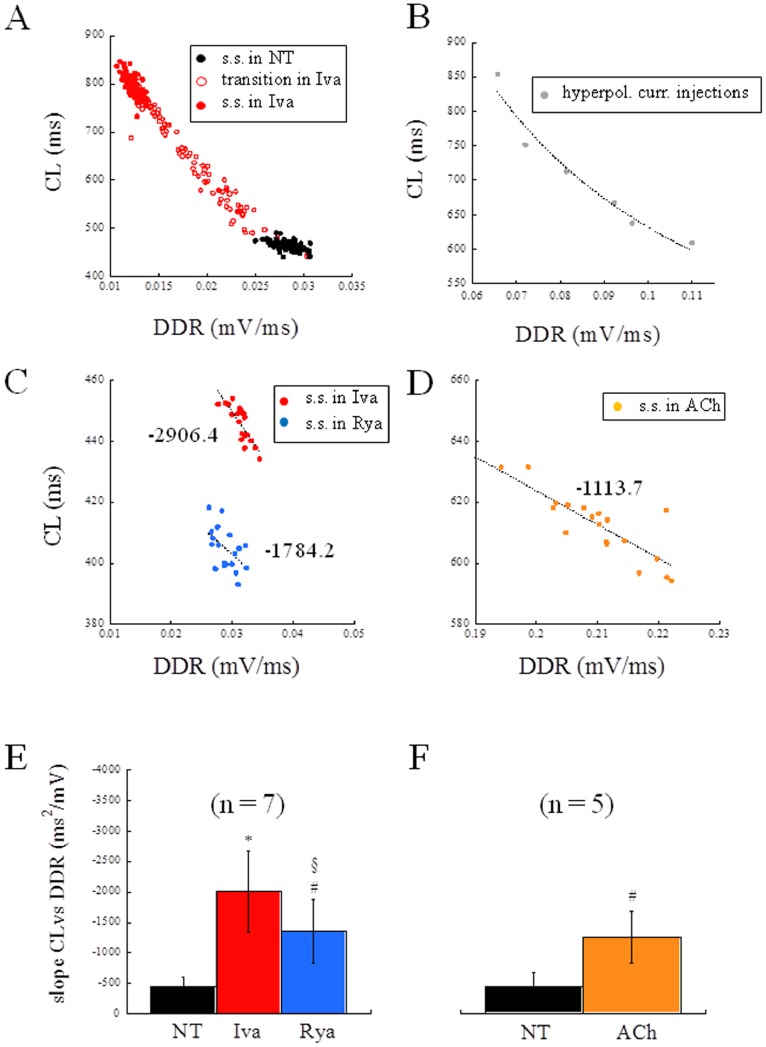
CL vs DDR relationship. Panel A: a representative example of hyperbolic relationship (R = 0.99) between CL vs DDR measured during application of Iva. Black dots refer to beats recorded at the steady state (s.s.) in NT; empty red dots refer to beats recorded right after Iva application; filled red dots refer to beats recorded at the s.s. in Iva. Panel B: CL vs DDR hyperbolic relationship (R = 0.98) measured from APs of the current injection experiment reported in Figure 8A. Panels C and D: steady state CL vs DDR relationships for Iva and Rya (C), and ACh (D) were always well fitted by linear regressions, whose slopes are reported in figure. Panels E and F: the histograms summarize changes induced on the slope of CL vs DDR by Iva and Rya (E) and by ACh (F). Statistics: paired t-test. p<0.05 vs NT (*); p<0.1 vs NT (#); p<0.1 vs Iva (§);

The non-linear (hyperbolic) relationship between CL and DDR shown by Rocchetti et al. [16] during ACh application to rabbit SANCs holds for all treatments adopted in the present study (see representative examples in Figure 9A and B). When focusing, as we do here, on steady state beating conditions, only a portion of the entire curve is considered, which can always be well fitted with a linear regression (representative examples in Figure 9C and D). The average slope of such regression was found to be (ms^2^/mV) −451, −2008, and −1359 for NT, Iva, and Rya perfusion respectively, when measured in all the 7 cells under study (Figure 9E). In order to consider only comparable bradycardic effects, among the 15 cells exposed to ACh, we then selected those (n = 5) whose steady state CL fell within ±2 SD from mean CL in Iva. On these cells, the mean value of the slope of CL vs DDR was −1252 (Figure 9F). These results show that all bradycardic agents lead to an increase in the slope of CL vs DDR, which is much larger in the case of Iva.

### Stochastic Events during DD

**Figure 10:**
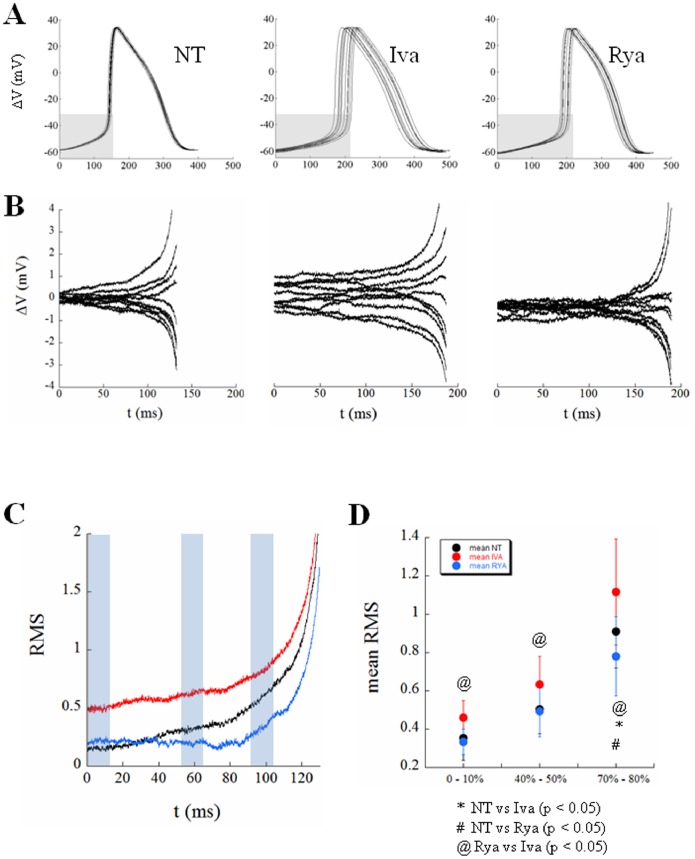
Beat-to-beat noise during DD. Panel A: Ten consecutive APs, time-aligned with the preceding MDP, are reported in each panel from a cell perfused, in turn, with NT, Iva, and Rya. Panel B: The DD phase (from MDP to TOP, corresponding approximately with the grey region in Panels A) was extracted from each curve and the average DD trace calculated for each treatment. The curves measured as the difference between each DD trace and the average DD trace are reported for each treatment. Panel C: The root mean square (RMS) of differences at each time was then calculated for NT (black trace), ivabradine (red), and ryanodine (blue), and all reported normalized to the duration of DD in NT. Panel D: RMSs of differences were then calculated for each one of the 7 cells under study, and the average results summarized for 3 different regions of DD, marked with light blue rectangles in Panel C, and reported in Panel D as mean ± SE.

A useful way to look at the variability of pacing rate is that reported in Figure 10. Panel A illustrates 10 superimposed consecutive APs, each one time-aligned with the preceding MDP, recorded in the three steady state conditions described above, i.e. under perfusion of NT, Iva, and Rya. We then extracted the DD phase of each 10 waveforms, taken from their MDP and TOP respectively (they fall approximately in the grey shaded rectangular area in each Panel of Figure 10A), calculated the average DD trace for each 10-beats sequence, and derived the corresponding difference traces, reported for the three treatments in Panel B. It appears that a certain degree of variation with respect to the average DD is intrinsically present in NT, increases with Iva, and decreases with Rya. This is best seen in the root mean square (RMS) derived for the three different groups of difference-traces and normalized with the time of the CL in NT (Panel C). There is a certain amount of beat-to-beat variation of DD with respect to the average DD in NT, which increases exponentially in proximity of the TOP; overall variation decreases during perfusion of Rya and remarkably increases during perfusion with Iva.

The same procedure was performed for the 7 cells and the average values collected in three ranges of DD (from 0 to 10%, from 40 to 50%, and from 70 to 80%, light blue rectangles in Panel C) reported as mean ± SE in Panel D. Whereas the difference between NT and Iva and that between NT and Rya did not always reach the statistical significance, that between Rya and Iva always did so. The cell whose results are reported in Panels A and B was chosen as representative of the overall statistical results of Panel D.

## Discussion

The rhythmic spontaneous electrical activity of cardiac SANCs is known to be sustained by two synergistic and coupled mechanisms, one related to the hyperpolarization-induced activation of the membrane ion current I_f_ (so called ‘membrane clock’), the other related to the inward Na^+^-Ca^2+^ exchanger current activated by sub-sarcolemmal LCRs (so called ‘calcium clock’) [23], [28]. Also, a long recognized property of SANCs firing is the intrinsic beat-to-beat CL variability of APs that they spontaneously exhibit [9] and that is expected to increase as mean CL does [16], [17]. The two clocks have been studied on small aggregates of rabbit SAN cells on the basis of the effects on several AP characteristics during rate modulation by means of Iva, a selective I_f_ blocker, and of Rya, which inhibits SR calcium release [28]. A clear involvement of the calcium clock in determining intrinsic CL variation of isolated rabbit SAN cells firing has been recently demonstrated [10]. Despite the relevance of the issue, information is still lacking on the relative contribution of the membrane and calcium clock in sustaining and modulating beat-to-beat CL variability of spontaneous APs generated by single SAN cells.

In the present study we specifically addressed this issue in order to test our hypothesis that the calcium clock, being intrinsically stochastic in its mechanism [10], would give a major contribution to beat-to-beat CL variability of SAN cells, as compared to the variability introduced by the membrane clock. In order to test this hypothesis, we patch clamped single guinea pig SANCs in whole-cell configuration, and modulated their firing rate by perfusing them, in turn, with 3 µM Iva, 3 µM Rya, 10 nM ACh, and 100 nM Iso.

The most striking result we found was that, despite the similar (not statistically different) bradycardic effect produced by Iva and Rya (∼30% increase in CL, cf. with +37% and +16% respectively found by Gao et al. in canine SANCs [33]), the two treatments led to a quite different variation in the level of beat-to-beat CL variability with respect to NT (Figure 3). CV_CL_ was 2.12% in normal conditions (NT), which is comparable to that shown by Wilders and Jongsma in single rabbit SAN cells (2.00%) and by Rocchetti et al. on the same preparation (2.30%) [9], [16]. Iva significantly increased CV_CL_ by 52% (by 100% in a larger sample), whereas Rya produced no significant change. The different effect of the two inhibitors on CL variability holds not only for relative changes (CV, %; Figure 4), but also for absolute ones (BVR, ms; Figure 5), despite the rather high dispersion of CL control (NT) values (see Table 1). This strengthens our observation, even in the absence of a significant difference in CL variability between Iva and Rya groups. The negligible presence of a longitudinal (along the bisecting line) component in our CL Poincarè plots (see representative example in Figure 5B) provides a further proof that AP sequences have been recorded in steady state pacing conditions [27], [31], [34]. Furthermore, all CL time series in NT were normally distributed (Figure 4C) and did not show significant autocorrelation within a lag 1 −20 beats (right Panel of Figure 2B), which also confirms the findings of Wilders and Jongsma in rabbit SAN cells. Both normal correlation and autocorrelation results hold during treatment with Iva and Rya, i.e. beat-to-beat changes in CL are randomly distributed and there is no apparent short-term memory involved in their time evolution, nor any average CL drift within the analyzed AP sequences. Similarly, normal distribution and absence of significant autocorrelation were found during ACh and Iso exposure (data not shown).

Although somewhat expected, changes in beat-to-beat variability of AP parameters other than CL are not strictly required in order to explain the increase in CV_CL_. From one hand, in fact, the system under study is over-determined with respect to AP trajectory, i.e. a certain phenotype (in our case a given CV_CL_) may correspond to a number of different parameters combinations [35], [36]. On the other hand, TOP depolarization (see top panel of Figure 3), for instance, can lead *per se* to an increase in CV_CL_, as the same extent of DDR variability can bring about such an increase when a more depolarized TOP has to be reached.

The increase in beat-to-beat CL variability during bradycardic interventions (including hyperpolarizing current injections), was expected from the known hyperbolic CL vs DDR relationship [16]. At slower beating rates, in fact, DDR gets smaller and the slope of CL vs DDR gets steeper, i.e. small changes in DDR lead to large CL changes on a beat-to-beat basis (see for instance Iva application in Figure 9A). When the calcium clock was inhibited, the beating rate slowed, and, following the decrease in mean DDR, the slope of CL vs DDR increased, as during ACh application (Figure 9E and F). The Rya-induced increase in the slope of CL vs DDR though, was not enough to bring about a significant increase in beat-to-beat CL variability. The much larger Iva-induced increase in slope led instead to the larger and significant increase in beat-to-beat CL variability that we document in the present study. This suggests that, when I_f_ functions as the main clock, the increase in CV_CL_ with CL follows the same law that regulates increase in CL variability during bradycardic interventions like current-induced hyperpolarization or muscarinic activation (Figure 8C and D). On the contrary, when the quasi-deterministic (mainly voltage-dependent) clock associated with whole-cell I_f_ is turned down, the intrinsic complex mechanism of the calcium clock can express all of its stochastic nature, leading to a significant increase in CV_CL_ (Figure 4 and red dot in Figure 8D) and reinforcing the view of a stabilizing role of I_f_ on SAN beating rate [37]. These stochastic fluctuations are present, as recently demonstrated [10], already at the very beginning of DD, but persist throughout this phase until the TOP (see Figure 10B, C, and D). A possible role of I_Kr_ inactivation in regulating pacemaker activity has been demonstrated in the past [38], [39] and could be involved in the observed Iva- and Rya-dependent APD prolongations. On the other hand, these were not accompanied by significant changes in MDP, nor in beat-to-beat APD variability, which has been shown to follow I_Kr_ inhibition in other cardiac cell types [32], [40], ruling out therefore a clear interplay between I_Kr_ and CL variability.

The present study describes for the first time the relative contribution of the two cardiac pacemaker clock mechanisms to the intrinsic variability of SANC beating rate. It would be tempting to explain the higher extent of CL variability induced by Iva application as a consequence of a much relevant decrease in total ionic current flowing through the membrane after I_f_ blockade. We have recently shown in a simulation study [41] that membrane resistance increases very much during SANCs DD; partial closure of I_f_ channels would make it even larger and lead therefore to a much higher sensitivity of AP trajectory to stochastic electrogenic LCRs of the calcium clock. We realize that this explanation is, at present, only speculative, and expect that simulations with SANC AP models including both membrane and calcium clocks [42] will better elucidate the mechanism.

Future investigations on more complex preparations, like isolated SA node or Langendorff perfused heart, could further dissect the relative contribution of the two clocks to the rate variability of SAN. The novel information provided here is, on the other hand, relevant in order to better understand the cellular control mechanism of physiological cardiac pacemaker rate, as well as to guide pharmacological research in developing new molecules for the fine regulation of cardiac rhythm and of its variability.
